# Conformation-Associated C···*d_z_*^2^-Pt^II^ Tetrel Bonding: The Case of Cyclometallated Platinum(II) Complex with 4-Cyanopyridyl Urea Ligand

**DOI:** 10.3390/ijms25074052

**Published:** 2024-04-05

**Authors:** Sergey V. Baykov, Eugene A. Katlenok, Svetlana O. Baykova, Artem V. Semenov, Nadezhda A. Bokach, Vadim P. Boyarskiy

**Affiliations:** Institute of Chemistry, Saint Petersburg State University, 7/9 Universitetskaya Nab., Saint Petersburg 199034, Russia; 195.08pt@gmail.com (E.A.K.); a.v.semenov@spbu.ru (A.V.S.); v.boiarskii@spbu.ru (V.P.B.)

**Keywords:** platinum metal complexes, tetrel bonding, stacking interactions, urea, pyridines, DFT

## Abstract

The nucleophilic addition of 3-(4-cyanopyridin-2-yl)-1,1-dimethylurea (**1**) to *cis*-[Pt(CNXyl)_2_Cl_2_] (**2**) gave a new cyclometallated compound **3**. It was characterized by NMR spectroscopy (^1^H, ^13^C, ^195^Pt) and high-resolution mass spectrometry, as well as crystallized to obtain two crystalline forms (**3** and **3**·2MeCN), whose structures were determined by X-ray diffraction. In the crystalline structure of **3**, two conformers (**3A** and **3B**) were identified, while the structure **3**·2MeCN had only one conformer **3A**. The conformers differed by orientation of the *N*,*N*-dimethylcarbamoyl moiety relative to the metallacycle plane. In both crystals **3** and **3**·2MeCN, the molecules of the Pt(II) complex are associated into supramolecular dimers, either {**3A**}_2_ or {**3B**}_2_, via stacking interactions between the planes of two metal centers, which are additionally supported by hydrogen bonding. The theoretical consideration, utilizing a number of computational approaches, demonstrates that the C···*d_z_*^2^(Pt) interaction makes a significant contribution in the total stacking forces in the geometrically optimized dimer [**3A**]_2_ and reveals the *d*_z_^2^(Pt)→π*(PyCN) charge transfer (CT). The presence of such CT process allowed for marking the C···Pt contact as a new example of a rare studied phenomenon, namely, tetrel bonding, in which the metal site acts as a Lewis base (an acceptor of noncovalent interaction).

## 1. Introduction

Deprotonated diaminocarbene complexes of late transition metals have been actively studied in the last decade [1,2,3]. From a practical point of view, these complexes have a lot of advantages. They effectively catalyze the processes of C–C cross-coupling [4,5,6,7,8], hydrosilylation [9,10,11], and C≡C triple-bond activation [12,13]. Moreover, several compounds are recognized as promising antitumor agents [14,15], probes of mercury(II) ions in solutions [16], and components of OLED devices [17].

Many studies demonstrate that a solid-state supramolecular structure plays a key role in reactivity [18] and photophysics [2,19,20,21] of such compounds. Their supramolecular organization is determined by combination of various noncovalent interaction: hydrogen bonding (HB) [22], π···π stacking [23], metallophilic interactions [24], halogen bonding [25], chalcogen bonding [26], pnictogen [27], and tetrel bonding (abbreviated as TtB) [28,29] etc. In recent years, special attention has been paid to interactions in which the metal center acts as a Lewis base (a nucleophile) due to a lone pair of electrons in the *d*_z_^2^ orbital [30]. Particularly, metal-involved halogen bonds [31,32,33,34,35,36], chalcogen bonds [37], spodium bonds [20], pnictogen bonds [38], and πh/C···M interactions [39,40,41,42,43] have been recognized. To contrast, in most examples of metal-involved TtB, a metal (commonly, tin or lead) is a σ- or π-hole donor, i.e., electrophilic site [44,45,46,47]. These interactions are the focus of many studies [48,49] due to importance of lead and tin coordination compounds for materials science [50,51,52,53]. At the same time, the TtB, in which the metal site acts as a Lewis base was only recently described. Particularly in our previous work, we experimentally and theoretically evidenced that the C···*d_z_*^2^(Pt) TtB can be a main component of metal-involving stacking interactions between half-lantern Pt^II^_2_ complexes and electron-deficient arenes [54,55].

In this work, we report a new example of C···*d_z_*^2^(Pt) TtB, which is responsible for supramolecular dimerization of one of two possible conformers of a deprotonated diaminocarbene Pt(II) complex in a solid state. To describe this TtB, X-ray diffraction data of the platinum complex and a combination of quantum chemical methods were used; all these results are given in the sections below.

## 2. Results and Discussion

### 2.1. Synthesis and Characterization of ***3***

**Scheme 1:**
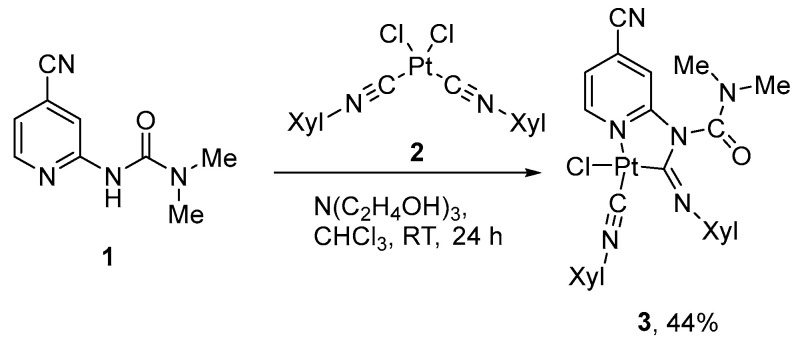
Synthesis of the cyclometallated complex **3** with 3-(4-cyanopyridin-2-yl)-1,1-dimethylurea ligand.

Target complex **3** (Scheme 1) was prepared according to the previously reported methodology [10,56]: the treatment of 3-(4-cyanopyridin-2-yl)-1,1-dimethylurea (**1**) with equivalent amounts of *cis*-[Pt(CNXyl)_2_Cl_2_] and triethanolamine in chloroform at room temperature (Scheme 1). The complex was studied using NMR (^1^H, ^13^C, ^195^Pt) spectroscopy and HR mass spectrometry. All obtained data are in a full agreement with the supposed structure and display common features of such compounds [15]. In particular, in the ^1^H and ^13^C NMR spectra, splitting of the signals of the methyl groups in the carbamoyl and xylyl moieties was observed, which indicates the double-bond character of the carbon–nitrogen bond. In the ^1^H spectra of both compounds, the α-CH protons of the pyridine rings appear as broad doublets (9.50 ppm), while in the starting urea **1** ^1^H spectrum the corresponding proton resonates at 8.31 ppm. In addition, the chemical shift of ^195^Pt for **3** (−3801 ppm) is close to those for other Pt(II) metallacycles with *N*-pyridylurea-based ligands (−3809–3802 ppm) [10,15].

### 2.2. Crystals and Their X-ray Structures

**Figure 1:**
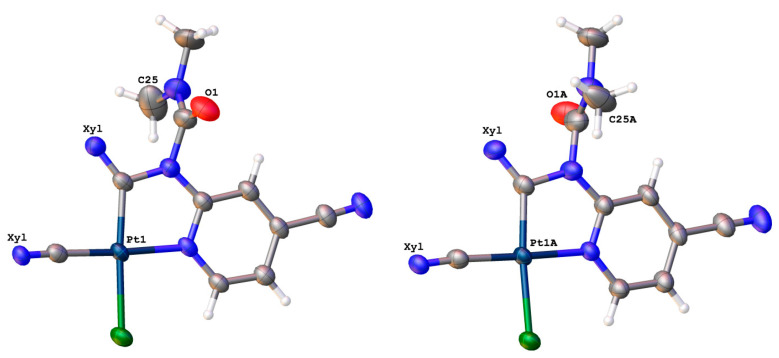
Two conformers **3A** (**left**) and **3B** (**right**) of the complex **3**, realized in its crystal structure. The xylyl moieties are omitted for simplicity.

The complex was crystallized from 1,2-dichloroethane and MeCN to give two different crystalline forms: **3** and **3·**2MeCN, the structures of which were determined by X-ray diffraction. The crystal structure of **3** includes two crystallographically independent molecules of the metal complex, differing in the orientation of the *N*,*N*-dimethylcarbamoyl moiety (Figure 1). Thus, these two types of molecules can be considered as two possible conformers of complex **3**, namely, **3A** (the carbamoyl moiety is turned by carbonyl group towards a viewer, Figure 1 Left) and **3B** (the carbamoyl moiety is turned by methyl group towards a viewer, Figure 1 Right). Notably, among four previously published examples of Pt(II) cyclometallated complexes with *N*-pyridylureas as a ligand [10,15], three structures (CSD refcodes: CAMPOZ, CAMQUG, PESLEI) represent type **A** conformers and one structure (CSD refcode CAMPIT) contains a type **B** conformer. In the structure of **3·**2MeCN, only conformer **3A** was revealed.

**Figure 2:**
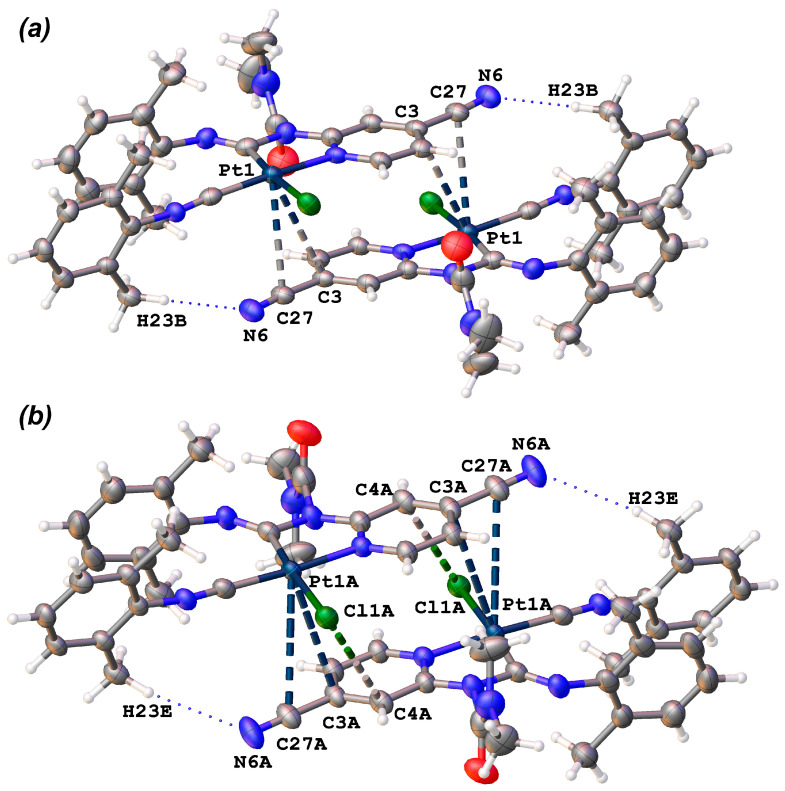
Structures of supramolecular dimers {**3A**}_2_ (**a**) and {**3B**}_2_ (**b**).

In both structures (**3** and **3·**2MeCN), the molecules of the metal complex are associated into supramolecular dimers via stacking interactions between two metal square-planes (each of which includes the 4-cyanopyridine moiety and the Pt(II)-based metallocycle, Figure 2 and Figure S1, Table 1 and Table 2), with additional supporting by several hydrogen bonds. In the structure of **3**, conformers **3A** and **3B** form two types of dimers ({**3A**}_2_ (Figure 2a) and {**3B**}_2_) (Figure 2b), in which the carbamoyl moieties are oriented relative to each other, like “C=O to C=O” ({**3A**}_2_) and like “CH_3_ to CH_3_” ({**3B**}_2_). In dimer {**3A**}_2,_ the molecules of the metal complex are located much closer to each other in comparison with {**3B**}_2_. Particularly in {**3A**}_2,_ the shortest interplanar distance is the distance between two pyridine rings (3.479(3) Å; the shortest C···C contact is 3.386(5) Å; Table 1) and it has an appropriate value for typical π···π stacking (3.41–3.61 Å) [57,58]. In contrast, in {**3B**}_2,_ the metal square-planes are more parallel displaced in comparison with {**3A**}_2_, which is illustrated by the “r” value (the distance between the centroid of one of the ring and the projection of the centroid of a π···π stacked ring to the first plane, Table 1). They are 0.948(5) Å and 1.805(6) Å for {**3A**}_2_ and {**3B**}_2,_ respectively. The interplanar distance in {**3B**}_2_ is significantly larger (3.987(3) Å) than in {**3A**}_2_. The shortest C···C contact in {**3B**}_2_ is 3.613(6) Å, which is at the end of the range of typical π···π stacking interactions (3.41–3.61 Å) [57,58]. According to our observation, this difference is caused by the steric repulsion of two methyl groups of the carbomoyl moieties facing each other in the case of the dimer {**3B**}_2_.

In the structure of **3·**2MeCN, conformer **3A** also provides the dimer with “C=O to C=O” arrangement of the carbamoyl moieties; however, its geometry is slightly different ({**3A’**}_2_, Figure S1). In this case, the shortest interplanar distance is between the pyridine ring and the metallacycle (3.547(2) Å; the shortest C···C contact is 3.275(5) Å), which also belongs to the range of typical π···π stacking interactions (3.41–3.61 Å) [57,58]. 

The metal centers in all dimers form contacts with the 4-cyanopyridine moieties (PyCN), namely, the C_CN_–C_Py_ bonds are located above the Pt atoms. The shortest Pt^II^-involved contacts are C_CN_···Pt (3.419(4) Å), C_Py_···Pt (3.414(3) Å), and C_Py_···Pt (3.677(4) Å) for {**3A**}_2_, {**3A’**}_2_, and {**3B**}_2_, respectively (Figure 2 and Figure S1, Table 2). In the case of {**3A**}_2_ and {**3A’**}_2_, both C···Pt distances are less than the sum of the Bondi vdW radii (*Σ*B_vdW_ (C + Pt) = 3.45 Å) [59,60], and all three C···Pt distances for {**3A**}_2_, {**3A’**}_2_, and {**3B**}_2_ are less than the sum of the Alvarez vdW radii (*Σ*A_vdW_ (C + Pt) = 4.06 Å) [61,62]. It should be mentioned that in the previous work [55], describing Pt···C tetrel bond, the C···Pt distances were larger that the corresponding *Σ*B_vdW_. Moreover, according to results of the early performed CSD search [55], the C···Pt contacts in our structures belong to top 15% of the shortest known C···Pt contacts. 

As it was mentioned above, the stacking interactions are additionally supported by hydrogen bonds (H_2_C–H···N for all dimers and C_Py_–H···O for {**3A**}_2_ and {**3A’**}_2_) (Figure 2). Geometrical parameters of these hydrogen bonds are collected in Table S3. Moreover, in the dimer {**3B**}_2,_ a C_Py_···Cl short contact was observed (3.579(4) Å vs. *Σ*BvdW [59] (C + Cl) = 3.45 Å and Alvarez radii sum *Σ*AvdW (C + Cl) = 3.59 Å) [61,62].

### 2.3. Theoretical Considerations of the Dimers [***3A***]_2_ and [***3B***]_2_

Several forces, including C···Pt TtB, C···Cl TtB, and π···π interactions, as well as C_Py_–H···O and H_2_C–H···N HBs, are responsible for the formation of supramolecular dimers {**3A**}_2_ and {**3B**}_2_. The nature of these interactions was studied theoretically by the DFT (PBE0-D3BJ) method. Since the crystal packing may significantly affect the intermolecular interactions, we carried out the geometry optimization of three isolated bimolecular clusters with starting geometries {**3A**}_2_ and {**3A’**}_2_ and {**3B**}_2_ and obtained two geometry optimized dimes abbreviated as [**3A**]_2_ and [**3B**]_2_. It should be noted that the geometry of the optimized dimer [**3A**]_2_ was the same regardless of the starting dimer ({**3A**}_2_ or {**3A’**}_2_) and only slightly differed from the starting X-ray dimers. The geometry-optimized dimer [**3B**]_2_ demonstrated changes that led to elongation of the C···Pt contact with simultaneous shortening of the C···Cl contact as compared to the X-ray dimer {**3B**}_2_.

The nature of C···Pt and C···Cl contacts that occurred between molecules of **3** was investigated by the wave analysis including the quantum theory of atoms in molecules (QTAIM) [63,64], independent gradient model based on Hirshfeld partition (IGMH) [65], electron localization function (ELF) [66], the charge displacement function (CDF) [67,68], methods combined with the extended transition state natural orbital for chemical valence theory (ETS–NOCV) [69], and natural bond orbital (NBO) [70] methods. Moreover, interaction energies were estimated according to super-molecular (SM) approach and the generalized Kohn–Sham energy decomposition analysis (GKS-EDA) [71].

#### 2.3.1. Molecular Electrostatic Potential

**Figure 3:**
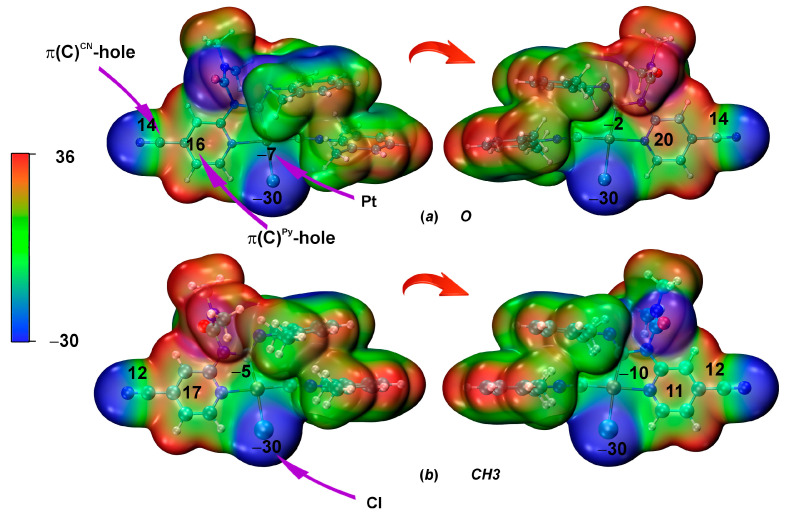
MEP distributions for the optimized structures of conformers **3A** (**a**) and **3B** (**b**); Vs,max and Vs,min values in kcal/mol, electron density isosurfaces 0.001 a.u. The potential values are given for the Pt, C, Cl atoms, as well as for the C of the pyridine ring.

As the first step of the theoretical study, the MEP of both conformers **3A** and **3B** was computed at the DFT (PBE0-D3BJ/ZORA-def2-TZVP) level of theory (**Figure 3**). Platinum atoms for both **3A** and **3B** are characterized by electronegative potential (Vs,min = (−6)–(−2) kcal/mol (**3A**) and (−10)–(−5) kcal/mol (**3B**)). For both conformers, two positive regions were revealed: first, on the center of the pyridine ring on both sides of the cycle (Vs,max = 16–20 kcal/mol (**3A**) and 11–17 kcal/mol (**3B**)) and second, a belt between the C and N atoms of the cyano group ring (Vs,max = 14 for **3A** and 12 kcal/mol for **3B**). Such belt is typical for cyano groups [72].

**Figure 4:**
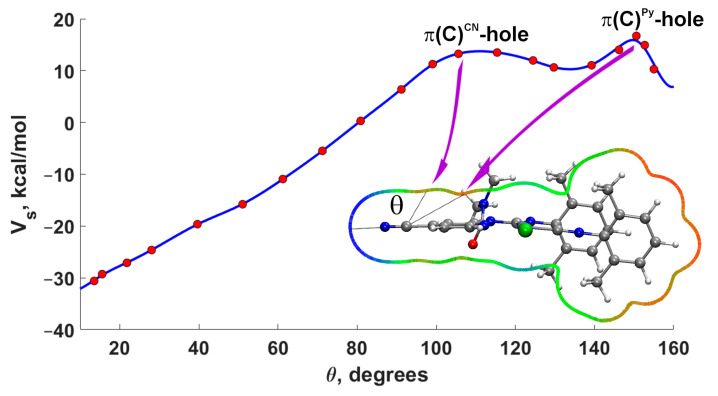
Dependence of the MEP value vs. angle θ for **3A** (0.001 a.u. isosurface of the electron density). The MEP plot is shown around the molecule in the plane passing through the CN atoms and the middle point of the C–C bond of the pyridine cycle (blue–green–red color scale −30 < V_s_ < 36 kcal mol^−1^).

For a more precise analysis of the presence of π-holes on carbon atoms, we constructed the dependence of the MEP (0.001) value on the angle in the plane passing through the CN group and the pyridine ring [73]. Examination of the plot (Figure 4) for **3A** indeed confirms the presence of maxima at angles of 108° and 150°. The first maximum corresponds to the π-hole on the carbon atom of the CN group, while the second maximum relates to the π-hole on the carbon atom of the pyridine ring. It should be noted that the π-hole is shifted towards the center of the ring, which is characteristic of aromatic rings [74]. 

#### 2.3.2. QTAIM and IGMH

**Figure 5:**
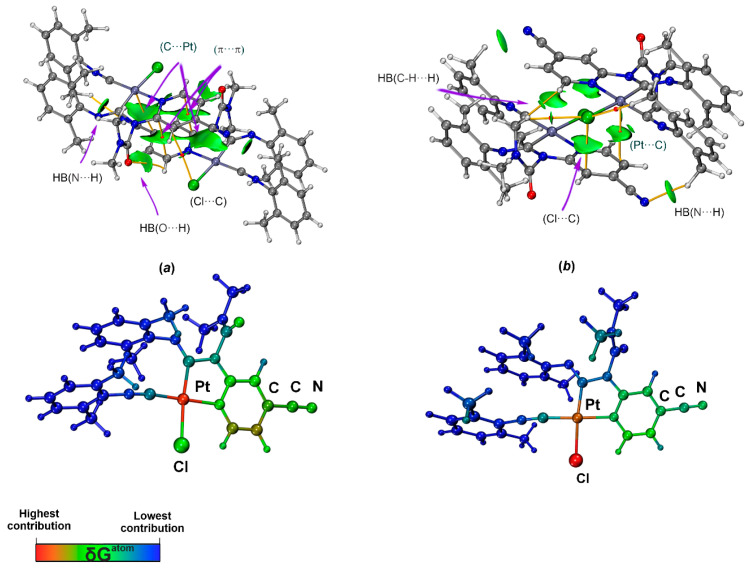
Sign(λ_2_)ρ(r) function mapped on the δg^inter^ isosurface (δg^inter^ = 0.006 a.u. and blue–cyan–green–yellow–red color scale −0.01 < sign(λ_2_)ρ(r) < 0.01; top) and the δG^atom^ colored structure (BCPs are shown as red dot, selected bond paths are shown as orange lines; bottom) for the structure of [**3A**]_2_ (**a**) and [**3B**]_2_ (**b**).

Analysis of both optimized bimolecular clusters ([**3A**]_2_ and [**3B**]_2_) using the QTAIM method allowed identification of bond critical points (BCPs, maxima of the density along bond paths) and bond paths, indicating interatomic interactions C···Pt, C···Cl, C···C, and HBs (Table 3 and Figure 5). Since the clusters are symmetrical, only unique contacts will be discussed in the manuscript to avoid a repetition. The small electron density values (ρ_b_ = 0.003–0.012 a.u.), positive Laplacian (∇2ρ_b_ = 0.012–0.042 a.u.), and positive total energy densities (Hb = 0.001–0.002 a.u.) indicate all these contacts are weak closed-shell noncovalent interactions. The electronic density at the BCPs for the C···Pt contact in [**3A**]_2_ is greater than in [**3B**]_2_, while the opposite trend is observed for the C···Cl contact. These results indicate that the C···Pt interaction is stronger in [**3A**]_2_, whereas the C···Cl interaction is stronger in [**3B**]_2_. The ELF value at the C···Pt and C···Cl bond critical points is 0.04, indicating efficient bonding between Pt or Cl and the C centers.

The QTAIM analysis also reveals the presence of various HBs that are involved in the association of **3A** and **3B** molecules into corresponding dimers. To estimate HBs energy we used the Espinosa–Molins–Lecomte formula [75] (E_int_(HB) ≈ 0.5V_b_). Values of HBs energy were calculated, with energies ranging from −0.5 to −2.1 kcal/mol. The HB energy values are given in the Table 3 for each dimer.

In addition, two BCPs and bond paths corresponding to C···C interactions, which confirm π···π stacking between the arene rings, were found in the calculated structure [**3A**]_2_. The ρ_b_ values indicate that these C···C interactions are weaker than the corresponding C···Pt contacts and HBs.

The IGMH isosurface for [**3A**]_2_ shows large areas of attractive interactions between the π-systems of two **3A** molecules, corresponding to the C···Pt and C···C contacts (Figure 5b). At the same time, for the C···Cl contact, the isosurface at values of δg(inter) = 0.006 is not detected, indicating a negligible contribution of C···Cl to dimer bonding. The investigation of the δG^atoms^ distribution [76] (Figure 5a) shows that the Pt atom indeed makes the largest contribution to the interactions between the π-systems, demonstrating the key role of the metal in supramolecular dimerization of **3A**. Negative sign(λ_2_)ρ(r) functions were also found between N or O atoms and H atoms, indicating intermolecular HB N···H and O···H.

For the IGMH isosurface (Figure 5b) of the [**3B**]_2_ dimer, green disk-shaped surfaces were found between the atoms (Pt or Cl) and C atom, confirming the presence of C···Cl and C···Pt contacts. However, the Cl atom dominates in dimer bonding, as evidenced by the δG^atom^ contribution distribution scheme. The presence of HB in the dimer structure is also confirmed by the IGMH method.

#### 2.3.3. ETC-NOCV/CDF and NBO

**Figure 6:**
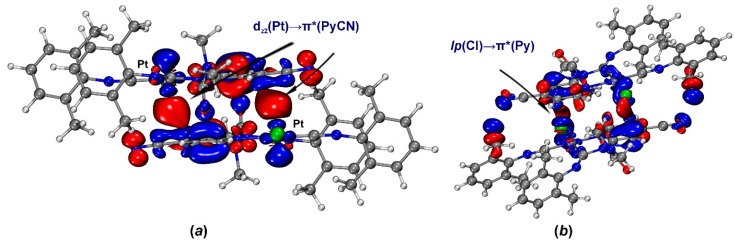
ETS-NOCV deformation densities for [**3A**]_2_ (**a**) and [**3B**]_2_ (**b**); isovalues 0.0005 a.u., electrons transfer occurs from the decreased electron density regions (blue) to the increased electron density regions (red).

The charge transfer (CT) effect is critical for categorizing a noncovalent interaction into a specific type. As has previously been shown, CT significantly contributes to the metal-involving TtB in cocrystals of [Pt(pbt)(μ-S^∩^N)]_2_ (S^∩^N = 2-thiopyridine, pbt = 2-phenylbenzothiazole) with electron-deficient arenes [55]. We analyzed the CT in [**3A**]_2_ and [**3B**]_2_ using the CDF and ETS-NOCV methods. ETS-NOCV enables estimation of individual orbital contributions to the electron density difference between isolated molecules and their resultant bimolecular cluster (Figure 6). Meanwhile, CDF visualizes and quantifies the CT at the moment of bond formation.

**Figure 7:**
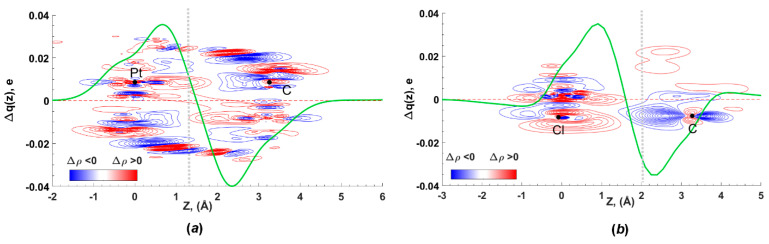
Total EDD contour plot (red—charge concentration, blue—charge depletion, range—0.01 to 0.04 a.u., step 0.0002 a.u.) and CDF functions for the C···*d_z_*^2^(Pt) interaction in [**3A**]_2_ (**a**) and the C···*lp*(Cl) interaction in [**3B**]_2_ (**b**); black dots indicate positions of the atomic nuclei, grey vertical lines identify the boundaries between the C, Pt, and Cl atoms, which are placed along the *z* axis.

According to ETS-NOCV data of [**3A**]_2_ (Figure 6a), *d*_z2_(Pt)→π*(PyCN) (PyCN is 4-cyanopyridine moiety) orbital interaction results in the electron density accumulation in the intermolecular space between fragments. This interaction provides 60% of the total energy of the orbital interaction (E_int_^orb^) between of two molecules of **3A** in the bimolecular cluster [**3A**]_2_. Analysis of the CDF curve for this pair of NOCV demonstrates the presence of the CT, which is estimated at 10 millielectron (m*e*) (Figure 7a). In other cases, orbital interactions associate with HBs and intramolecular polarization in the absence of effective intermolecular CT. The involvement of charge transfer with platinum is also confirmed by the increase in Hirshfeld charges charge on the Pt atom by 4 m*e*.

**Figure 8:**
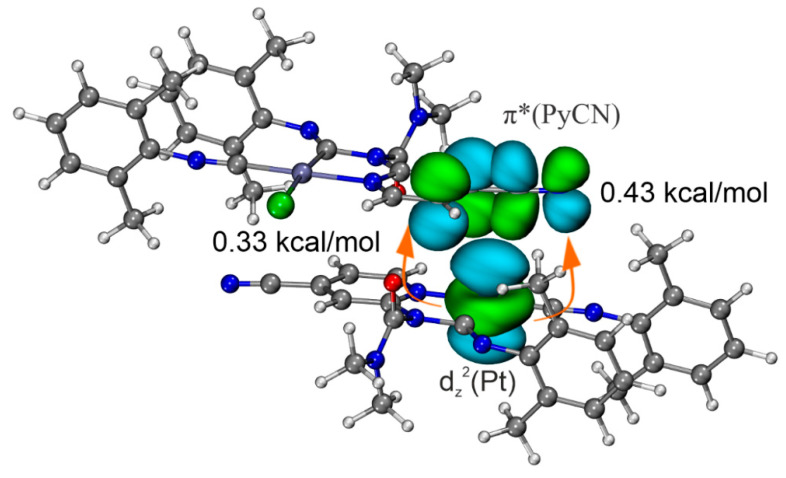
Natural bond orbitals corresponding to the *d*_z_^2^(Pt)→π*(PyCN) lone pair transitions in [**3A**]_2_; PyCN is a 4-cyanopyridine moiety.

Second-order perturbation theory (E2), based on NBO analysis, also reveals the presence of *d*_z_^2^(Pt)→π*(PyCN) CT. NBO analysis shows that the direct CT in the C···Pt contact is associated with lone pair transitions from *d*_z_^2^(Pt) to π*-orbital of PyCN moiety with total second-order perturbation energies E(2) −0.76 kcal/mol (Figure 8). Thus, the *d*_z_^2^(Pt)→π*(PyCN) CT is an important component of the studied C···*d_z_*^2^(Pt) interaction, and, therefore, this noncovalent contact should be attributed to TtB.

NOCV results indicate significant orbital interactions (50% of the total E_int_^orb^) between the Cl atom and π*(PyCN) for the dimer [**3B**]_2_. According to the CDF curve, the charge transfer amounts to 28 m*e*, considerably exceeding the analogous value for the [**3A**]_2_ dimer (Figure 7b). This indicates pronounced CT from the chloride orbitals to the π*(PyCN) orbital. The Hirshfeld charges on the chlorine atom decrease from −0.328*e* to −0.309*e*, while the charges on the platinum atom remain practically unchanged. Due to the CT, the C···Cl interaction in the [**3B**]_2_ dimer satisfies the criteria of a TtB. In contrast to the dimer [**3A**]_2_, the *d*_z_^2^(Pt)→π*(PyCN) orbital interaction is absent in the dimer [**3B**]_2_. The HB, orbital polarization, and intramolecular electron distribution are responsible for the residual part of the total E_int_^orb^.

**Figure 9:**
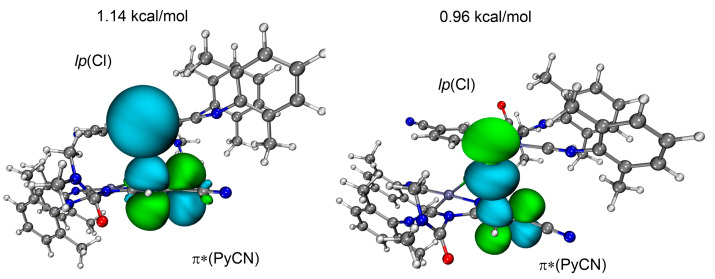
Natural bond orbitals corresponding to the *lp*(Cl)→π*(PyCN) lone pair transitions in [**3B**]_2_; PyCN is a 4-cyanopyridine moiety.

NBO analysis results also emphasize the definitive role of the chloride ligand in enabling orbital interactions between complexes in the [**3B**]_2_ dimer. The total energy of the *lp*(Cl)→π*(PyCN) donor–acceptor interaction is 2.1 kcal/mol, exceeding the analogous value for *d*_z_^2^(Pt)→π*(PyCN) in the dimer [**3A**]_2_ (Figure 9). Thus, in the dimer [**3A**]_2,_ the *d*_z_^2^(Pt)→π*(PyCN) interaction mediated by charge transfer is dominant, while in the [**3B**]_2_ dimer, the *lp*(Cl) interaction with π*(PyCN) prevails over the analogous *d*_z_^2^(Pt)→π*(PyCN) interaction, serving as the primary center of orbital interactions. At the same time, the C···*d_z_*^2^(Pt) interaction in the [**3B**]_2_ cannot be classified as a tetrel bond due to the lack of CT involving platinum. Based on the significant charge transfer, the *d*_z_^2^(Pt)→π*(PyCN) interaction in the dimer [**3A**]_2_ and the *lp*(Cl)→π*(PyCN) interaction in the dimer [**3B**]_2_ can be attributed to tetrel bonds.

#### 2.3.4. Interaction Energies 

**Figure 10:**
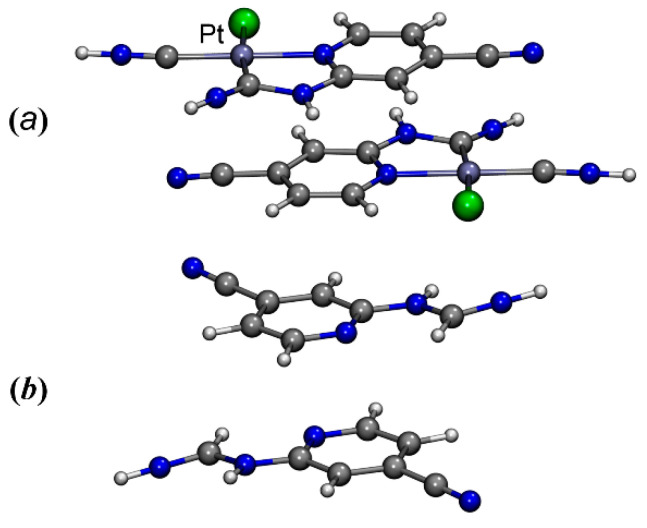
Model bimolecular clusters (**a**) M[**3A**]_2_ and (**b**) M1[**3A**]_2_.

The overall interaction and binding (*Σ*E_int_^SM^ and *Σ*E_b_^SM^) energies for the formation of bimolecular clusters [**3A**]_2_ and [**3B**]_2_ were calculated using the SM approach. The corrected interaction energies for the BSSE error are given in the Table 4. These energies have values of more than 20 kcal/mol, which correspond to strong noncovalent interaction. As indicated in Section 2.3.2, two types of noncovalent force are responsible for the association of **3A** into the bimolecular cluster [**3A**]_2_, i.e., the stacking interaction (with participation of C···*d_z_*^2^(Pt) TtB) between two planes, each of which consists of the 4-cyanopyridine moiety and the Pt(II)-based metallocycle, and several HBs. In order to quantitatively estimate the energy of the stacking interaction and C···*d_z_*^2^(Pt) TtB in [**3A**]_2_, interaction energy calculations were performed on specifically constructed model systems (M[**3A**]_2_ and M1[**3A**]_2_; (Figure 10a). To eliminate the influence of auxiliary HBs, in the first simplified model of the M[**3A**]_2_ the dimethylcarbamoyl and xylyl moieties were replaced with hydrogen atoms located at a distance of 1 Å. In the second model of the M1[**3A**]_2_, to eliminate the coordination bond with the metal, the {PtClCNH} fragment was removed from the M[**3A**]_2_ structure (Figure 10b), which made it possible to calculate the energy of the pure π···π interaction between the aromatic systems. Thus, by subtracting the interaction energy of the second model from the first one, it is possible to estimate the contribution of the tetrel bond C···*d_z_*^2^(Pt) (according to the formula: E_int_(C···*d_z_*^2^(Pt))^SM^= (E_int_ (M [**3A**]_2_^SM^) − E_int_(M1[**3A**]_2_)^SM^)). The obtained values of the energies of π···π stacking and C···*d_z_*^2^[Pt] were −12 kcal/mol and −5 kcal/mol, respectively. The remaining part of the energy −9.2 kcal/mol is accounted for by intermolecular hydrogen bonds, which is consistent with the results of estimating their energy by the QTAIM method (Section 2.3.2). Thus, the contribution of the C···*d_z_*^2^(Pt) TtB interaction accounts for 45% of the total interaction energy for the dimer [**3A**]_2_, proving the important role of tetrel bonding interactions with the metal in the association of the complex **3**.

A similar calculation procedure was also conducted for the second dimer, except that the C···lp(Cl) interaction energies were calculated instead of C···Pt (according to the formula: E_int_(C··· lp(Cl))^SM^ = (E_int_(M [**3B**]_2_^M^) − E_int_(M1[**3B**]_2_)^SM^)). It can be concluded that the TtB C···lp(Cl) interaction in the second dimer is the dominant one and accounts for 66% of the total interaction energy between the molecules **3B** in the dimer. In comparison, in the first dimer the contribution of the TtB C···Pt interaction was 45% of the total binding energy. Thus, the results of the model system energy calculations demonstrate the significance of both platinum and chlorine as nucleophilic centers providing intermolecular interaction through the formation of TtBs with the carbon of the pyridine ligand. Both kinds of interactions play an important role and are a determining factor in the dimerization of the complexes in the considered dimer types.

Finally, we applied the GKS-EDA method [71] to M[**3A**]_2_ and M[**3B**]_2_ in order to gain further insight into the nature of the studied interaction (Table 5). In both dimers, we observe a similar composition of energy components. The GKS-EDA results indicate the predominantly dispersive character of the attractive interactions, accounting for 54% and 51% of the total attractive energy in M[**3A**]_2_ and M[**3B**]_2_, respectively. The electrostatic component makes the second largest contribution to the attractive energy, comprising 34% and 38% of the total attractive interaction in M[**3A**]_2_ and M[**3B**]_2_, respectively. The polarization component, although relatively insignificant and amounting to only 12% and 11%, however, plays a stabilizing role in the attractive interaction in M[**3A**]_2_ and M[**3B**]_2_, respectively.

The GKS-EDA results underscore dispersion as the predominant source of attractive interactions that stabilize the dimerization of the studied conformers of the complex **3**. The comparable energetic decomposition for bimolecular model M[**3A**]_2_ and M[**3B**]_2_ further evinces the analogous interaction mechanisms for dimers connected through C···*d_z_*^2^(Pt) and C···*lp*(Cl) TtBs.

## 3. Conclusions

We found the Pt(II) complex **3** bearing deprotonated 3-(4-cyanopyridin-2-yl)-1,1-dimethylurea as a ligand was crystallized in form of two conformers **3A** and **3B**, owing to different orientation of the *N*,*N*-dimethylcarbamoyl moiety, in the structure of **3**, and only one conformer **3A** in **3**·2MeCN. Independently on the type of conformer, molecules of **3** are associated into the “plane-to-plane” supramolecular dimers, {**3A**}_2_ or {**3B**}_2_, correspondingly. The stacking interaction between two metal square-planes, each of which consists of the 4-cyanopyridine moiety and the Pt(II)-based metallocycle, as well as hydrogen bonding, are responsible for the formation of these dimers. In its turn, the stacking interaction includes several components, namely, a π···π interaction between the heteroaromatic rings and a C···*d_z_*^2^(Pt) contact between the carbon atom of the substituted 4-cyanopyridyl moiety and the metal center. The contact C···*d_z_*^2^(Pt) in {**3A**}_2_ was attributed to metal-involving TtB, in which the metal site acts as a Lewis base (an acceptor of noncovalent interaction). This suggestion is based on structural parameters and quantum chemical calculation data for the optimized dimer [**3A**]_2_. Particularly, there is (1) the presence of a C···Pt short interatomic distance in the X-ray crystal structure; (2) the presence of a bond critical point and bond path in the QTAIM analysis; (3) the presence of a π-hole on the nitrile carbon atom and pyridine ring (as pronounced local maxima on the MEP surface); (4) the presence of significant charge transfer (more than 5 m*e*) from the nucleophile’s orbital to the vacant π-orbital involving the π-hole of the electrophile (the carbon atom); (5) negative value of the interaction energy. A similar example of metal-involving TtB was previously described for cocrystals of a binuclear platinum(II) complex with perfluorinated aromatic compounds [55]. In both these cases, the platinum(II) centers behave as TtB acceptors, which becomes possible due to the enhanced nucleophilicity of this metal center. In contrast, the X-ray dimer {**3B**}_2_ has different geometry to the corresponding optimized dimer [**3B**]_2_: the C···Pt contact is lengthened and weakened after optimization, and the optimized dimer [**3B**]_2_ exhibits C···*lp*(Cl) TtB as one of the strongest noncovalent forces, which was confirmed theoretically.

The most significand finding of this work is the TtB C···*d_z_*^2^(Pt) in {**3A**}_2_. We believe that new example of a metal-involving TtB will significantly expand understanding the phenomenon of noncovalent interactions involving a transition metal as a Lewis base.

## 4. Experimental Section

### 4.1. Materials and Instrumentation

3-(4-Cyanopyridin-2-yl)-1,1-dimethylurea (**1**) [77] and *cis*-[PtCl_2_(CNXyl)_2_] **2 [78]** were synthesized according to the literature protocols. All other reagents and solvents were purchased and were used as received in BLDPharm (Shanghai, China), Macklin (Shanghai, China).

^1^H, ^13^C, and ^195^Pt NMR spectra were recorded on a Bruker AVANCE III 400 spectrometer operating at room temperature at 400, 101, and 86 MHz for ^1^H, ^13^C, and ^195^Pt NMR spectra, respectively. All spectra were registered using CDCl_3_ as a solvent. The chemical shifts are given in δ-values (ppm). Multiplicities are abbreviated as follows: s = singlet, d = doublet, t = triplet, m = multiplet, br = broad; coupling constants, *J*, are reported in Hertz (Hz). High-resolution mass spectra (HRMS) were measured on Bruker Maxis HR-MS-ESI-qTOF using ESI. The most intense peak in the isotopic pattern is reported.

### 4.2. Synthesis of Complex ***3***

Triethanolamine (18 mg, 0.12 mmol) was added to a mixture of the urea **1** (19 mg, 0.10 mmol) and *cis*-[PtCl_2_(CNXyl)_2_] **2** (81 mg, 0.12 mmol) in CHCl_3_ (3 mL). The reaction mixture was stirred at RT for 24 h. After that, the reaction mixture was filtered to remove a small amount of undissolved material (triethanolamine hydrochloride) and evaporated to dryness at 45 °C in vacuo. Complex **3** was purified by reprecipitation from dichloromethane. It was dissolved in dichloromethane (0.3 mL) and diluted with MeOH (1.1 mL). The formed precipitate was collected by filtration, washed with hexane, and dried in vacuo at RT. Light yellow powder; 44% yield (30 mg). ^1^H NMR (400 MHz, CDCl_3_): δ 9.50 (dd, *J* = 6.3, 0.8 Hz, 1H), 7.18–7.09 (m, 3H), 6.99 (d, *J* = 7.6 Hz, 2H), 6.78 (d, *J* = 7.5 Hz, 1H), 6.64 (d, *J* = 7.5 Hz, 1H), 6.17 (t, *J* = 7.5 Hz, 1H), 3.22 (s, 3H), 3.16 (s, 3H), 2.25 (s, 6H), 2.22 (s, 3H), 2.19 (s, 3H). ^13^C NMR (101 MHz, CDCl_3_): δ 157.1, 153.1, 150.4, 148.7, 147.7, 134.5, 129.2, 128.4, 128.0, 127.5, 127.4, 127.0, 124.9, 123.5, 115.9, 115.4, 111.5, 38.4, 36.7, 19.6, 19.3, 18.4. ^195^Pt NMR (86 MHz, CDCl_3_): δ −3801. HRMS (ESI) *m/z* [M–Cl]^+^ calculated for [C_27_H_27_ClN_6_OPt–Cl]^+^ 646.1889; found 646.1904.

### 4.3. Crystal Growth, Structure Solution and Refinement Details

Crystals **3** and **3·**2MeCN were grown by slow evaporation of solutions of the corresponding compound in 1,2-dichloroethane (**3**) or acetonitrile (**3·**2MeCN) in air at RT. X-ray diffraction data were collected at a Rigaku XtaLAB Synergy–S diffractometers using Cu-Kα (λ = 0.154184 nm) radiation. The structures have been solved with the ShelXT [79] structure solution program using Intrinsic Phasing and refined with the ShelXL [80] refinement package incorporated in the OLEX2 program package [81] using Least Squares minimization. Supplementary crystallographic data have been deposited at Cambridge Crystallographic Data Centre: 2323847 (**3**) and 2323848 (**3·**2MeCN). They can be obtained free of charge via www.ccdc.cam.ac.uk/data_request/cif (accessed on 14 February 2024).

### 4.4. Computational Details

Full geometry optimization of the model clusters has been carried out at the DFT level of theory using the PBE0 [82,83] functional with the atom-pairwise dispersion correction with the Becke–Johnson damping scheme (D3BJ) [84,85]. The ORCA package (version 5.0.3) was used for the calculation [86,87]. Zero-order regular approximation (ZORA) [88] was employed to account the relativistic effects. The X-ray structures (**3** and **3·**2MeCN) were used as initial geometries for the optimization procedure. The ZORA-def2-TZVP(–f) basis set was applied for the H, C, N, O, and Cl atoms, whereas the SARC-ZORA-TZVP basis set was used for the Pt atom [89]. The Hessian matrix was calculated analytically for the optimized structures to prove the location of correct minima (no imaginary frequencies). Combination of the “resolution of identity” and the “chain of spheres exchange” algorithms (RIJCOSX) [90] in conjunction with the auxiliary basis sets SARC/J were used [91]. Large integration grid (DEFGRID3) was used throughout the calculations.

The single point calculations based on the equilibrium geometries were performed at the PBE0-D3BJ level with the ZORA-def2-TZVP(–f) [88] (for H, C, N, O, and Cl) and the SARC-ZORA-TZVP (for Pt) basis sets [89]. This level of theory was used for the estimates of interaction energies at the super-molecular approach and for the QTAIM, ELF, IGMH, MEP, NBO, CDF, and ETS-NOCV analyses. The natural bond orbital analysis was performed using the NBO 7.0 program [70].

The QTAIM, ELF, IGMH, and MEP calculations were carried out using the Multiwfn 3.8 software [76,92,93] and results were visualized using the VMD program [94]. The generalized Kohn–Sham energy decomposition analysis (GKS-EDA) [71,95] is conducted at the PBE0-D3BJ/def2-TZVP level of theory by a local version of GAMESS-US (version 2021-R2 patch 1) [96,97]. The CDF and ETS-NOCV analyses were carried out according to the methodology described in refs. [68,98,99] using the Multiwfn 3.8 software. Basis set superposition error (BSSE) was estimated using the counterpoise method [100].

The interaction and binding energies between complex (E_int_ and E_b_) were calculated for bimolecular clusters as

E_int_(**3A**···**3A**) = E([**3A**]_2_) − 2E{**3A**} + BSSE, (1)

E_b_(**3A**···**3A**) = E([**3A**]_2_) − 2E(**3A**) + BSSE, (2)

where E([**3A**]_2_), and E(**3A**) are total energies of the optimized structures of [**3A**]_2_ and **3A**, while E{**3A**} are total energies of **3A** in the optimized geometry of [**3A**]_2_.

## Figures and Tables

**Table 1:**
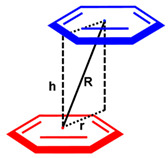
Geometrical parameters of the π···π stacking interactions in crystal structures of **3** and **3·**2MeCN.

Dimer	R, Å ^a^	h, Å ^b^	r_,_ Å ^c^	φ, ° ^d^	θ, ° ^e^
{**3A**}_2_	3.479(3)	3.348(3)	0.948(5)	0.0(4)	0.0(4)
{**3B**}_2_	3.987(3)	3.555(4)	1.805(6)	0.0(6)	0.0(5)
{**3A’**}_2_	3.668(3)	3.283(3)	1.635(5)	0.0(6)	0.0(4)
[**3A**]_2_^f^	3.406	3.236	1.063	0.2	0.4
[**3B**]_2_^f^	4.762	3.409	3.325	0.01	0.01

^a^ R is a distance between the centroid of one ring and the centroid of π···π stacked ring; ^b^ h is a distance between the centroid of one ring and the plane of a π···π stacked ring; ^c^ r is a distance between the centroid of one ring and the projection of the centroid of a π···π stacked ring to the first plane (rings offset); ^d^ φ is a twist angle is defined as the angle between the plane of the anchoring ring and the plane containing six atoms of pyridine ring; ^e^ θ is an angle between planes of adjacent π···π stacked ring; ^f^ herein and below, parameters for optimized bimolecular clusters [**3A**]_2_ and [**3B**]_2_ (given with square brackets), see Section 2.3.

**Table 2:** Geometrical parameters of the observed C···Pt contacts in supramolecular dimers {**3A**}_2_, {**3A’**}_2_, {**3B**}_2_.

Dimer	Contact	d(C···Pt), Å	Nc ^a^	
			Bondi	Alvarez
{**3A**}_2_	C3···Pt1	3.527(3)	1.02	0.87
	C27···Pt1	3.419(4)	0.99	0.84
{**3B**}_2_	C3A···Pt1A	3.677(4)	1.07	0.91
	C27A···Pt1A	3.848(4)	1.12	0.95
{**3A’**}_2_	C3···Pt1	3.414(3)	0.99	0.84
	C27···Pt1	3.633(3)	1.05	0.89
[**3A**]_2_	C3···Pt1	3.36	0.97	0.83
	C27···Pt1	3.42	0.99	0.84
[**3B**]_2_	C3···Pt1	3.77	1.09	0.93
	C27···Pt1	4.48	1.30	1.10

^a^ Herein and below, the “normalized contact” (Nc) is the ratio between the observed C···Pt distances and the sum of van der Waals radii of involved atoms, *Σ*B_vdW_ (C + Pt) = 3.45 Å [59,60]; *Σ*A_vdW_ (C + Pt) = 4.06 Å [61,62].

**Table 3:** Electron density (ρ_b_), its Laplacian (∇^2^ρ_b_), potential and kinetic energy densities (V_b_ and G_b_), second eigenvalue of the Hessian matrix (λ_2_) (in a.u.), electron localization function at BCPs calculated.

Cluster	Contact	ρ_b_	∇^2^ρ_b_	V_b_	G_b_	H_b_	λ_2_	ELF	Eint(HB)
[**3A**]_2_	C3···Pt1	0.0098	0.0283	−0.0051	0.0061	0.0010	−0.0026	0.0427	-
	C1···C5	0.0062	0.0208	−0.0031	0.0042	0.0010	−0.0011	0.0203	-
	H2···O1	0.0084	0.0340	−0.0050	0.0067	0.0018	−0.0049	0.0210	−1.6
	H23···N6	0.0098	0.0345	−0.0053	0.0070	0.0017	−0.0091	0.0334	−1.7
	C4···Cl1	0.0050	0.0166	−0.0023	0.0032	0.0009	−0.0008	0.0170	-
[**3B**]_2_	C33···Pt1A	0.0079	0.0204	−0.0037	0.0044	0.0007	−0.0020	0.0400	-
	C4A···Cl1A	0.0091	0.0276	−0.0045	0.0057	0.0012	−0.0039	0.0384	-
	H22F···Cl1A	0.0035	0.0102	−0.0015	0.0020	0.0005	−0.0018	0.0127	−0.5
	H25E ···Cl1A	0.0059	0.0193	−0.0028	0.0038	0.0010	−0.0033	0.0199	−0.9
	H25E···H1A	0.0063	0.0215	−0.0034	0.0044	0.0010	−0.0045	0.0190	−1.1
	H23E···N6A	0.0121	0.0418	−0.0067	0.0086	0.0019	−0.0114	0.0437	−2.1

**Table 4:** Calculated interaction and binding energies (E_int_ and E_b_, respectively).

	[3A]_2_	[3B]_2_
E_int_^SM^	−26.5	−25.0
E_int_^SM^(M)	−17.0	−20.1
E_int_^SM^(M1)	−5.3	−3.6
E_b_^SM^	−21.3	−20.9

**Table 5:** Calculated energy interaction and E_int_ decomposition (all in kcal/mol).

Clusters	ΔE^Ele^	ΔE^Paule^	ΔE^Pol^	ΔE^Disp^	ΔE_int_
M[**3A**]_2_	−17.0	31.7	−5.7	−26.7	−17.0
M[**3B**]_2_	−17.3	25.1	−4.9	−23.1	−20.2

## Data Availability

Data are contained within the article and Supplementary Materials.

## Supplementary Materials

The following supporting information can be downloaded at: https://www.mdpi.com/article/10.3390/ijms25074052/s1. X–ray diffraction data; Copies of NMR and HRMS spectra of complex 3; Computational study; Cartesian coordinates for the studied molecules.
