# Supplementary material for: Conformation-Associated C···dz2-PtII Tetrel Bonding: The Case of Cyclometallated Platinum(II) Complex with 4-Cyanopyridyl Urea Ligand
Source: Int J Mol Sci. 2024 Apr 5;25(7):4052. doi: 10.3390/ijms25074052 (PMC11012616; doi:10.3390/ijms25074052)
Supplement: Supplementary file 1 [file ijms-25-04052-s001.zip › ijms-2900453-supplementary.pdf]

Electronic supplementary materials

Conformation-Associated C···$d_z{}^2$-Pt^{II} Tetrel Bonding: The Case of Cyclometallated Platinum(II) Complex with 4-Cyanopyridyl Urea Ligand

Sergey V. Baykov*, Eugene A. Katlenok, Svetlana O. Baykova, Artem V. Semenov, Nadezhda A. Bokach* and Vadim P. Boyarskiy

Institute of Chemistry, St. Petersburg State University, 7/9 Universitetskaya Nab., Saint Petersburg 199034, Russia

Content

1. X–ray diffraction data..3

2. Copies of NMR and HRMS spectra of complex **3**...6

3. Computational study...8

4. Cartesian coordinates for the studied molecules 9

1. X–ray diffraction data

Table S1. Crystal data and structure refinement parameters for crystal **3** and **3·2**MeCN.

Cocrystal	**3**	**3**·2MeCN
Identification code	BSC-356	BSC-559
CCDC number	2323847	2323848
Empirical formula	$C_{27}H_{27}ClN_6OPt$	$C_{31}H_{33}ClN_8OPt$
Formula weight	682.08	764.19
Temperature, K	100(2)	100(2)
Crystal system	triclinic	triclinic
Space group	P-1	P-1
a, Å	14.2846(3)	9.87870(10)
b, Å	14.6219(2)	13.0012(2)
c, Å	16.4653(3)	13.0618(2)
α, °	89.2090(10)	77.0320(10)
β, °	69.989(2)	78.1850(10)
γ, °	84.4130(10)	89.0880(10)
Volume, $Å^3$	3215.40(11)	1599.38(4)
Z	4	2
ρ_{calc}g, cm^3	1.409	1.587
μ, mm^{-1}	9.134	9.271
F(000)	1336.0	756.0
Crystal size, mm^3	0.16 × 0.14 × 0.13	0.06 × 0.05 × 0.01
Radiation	Cu Kα (λ = 1.54184)	Cu Kα (λ = 1.54184)
2Θ range for data collection, °	5.714 to 139.994	6.98 to 159.906
Index ranges	$-16 \le h \le 17$, $-17 \le k \le 17$, $-19 \le l \le 20$	$-12 \le h \le 12$, $-16 \le k \le 16$, $-16 \le l \le 16$
Reflections collected	88421	60518
Independent reflections	12186 [R_{int} = 0.0770, R_{sigma} = 0.0349]	6745 [R_{int} = 0.0589, R_{sigma} = 0.0257]
Data/restraints/parameters	12186/0/661	6745/0/387
Goodness–of–fit on F^2	1.082	1.128
Final R indexes [I>=2σ (I)]	R_1 = 0.0318, wR_2 = 0.0869	R_1 = 0.0284, wR_2 = 0.0764
Final R indexes [all data]	R_1 = 0.0350, wR_2 = 0.0891	R_1 = 0.0293, wR_2 = 0.0771
Largest diff. peak/hole/ $eÅ^{-3}$	2.02/–1.73	1.22/–1.46

Table S2. Selected bond lengths and angles for complex **3** in obtained crystal structures.

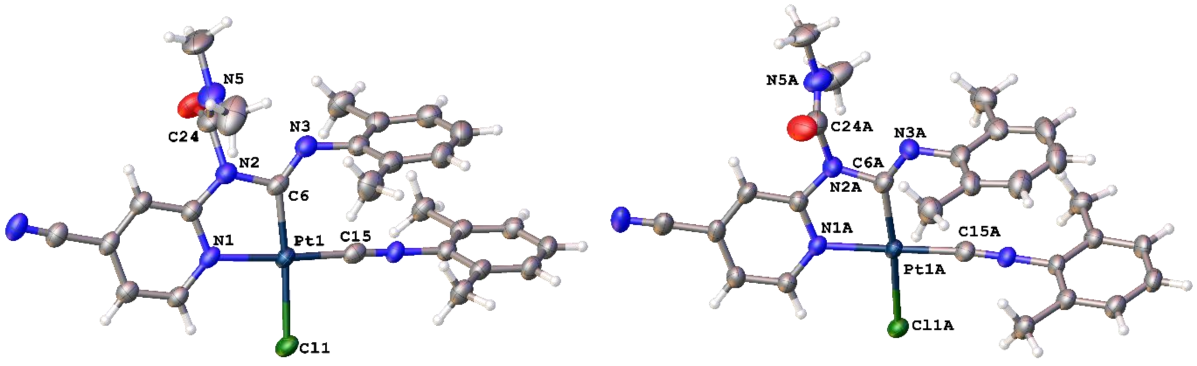

Parameter	**3**		**3**·2MeCN
Pt1–Cl1, Å	2.3801(11)	2.3770(8)	2.3787(9)
Pt1–N1, Å	2.051(3)	2.045(3)	2.050(3)
Pt1–C6, Å	1.992(4)	2.002(4)	1.924(4)
Pt1–C15, Å	1.986(4)	1.921(4)	1.924(4)
C6–N2, Å	1.433(5)	1.438(5)	1.467(6)
C6–N3, Å	1.265(5)	1.254(5)	1.266(4)
C24–N2, Å	1.467(6)	1.458(4)	1.478(6)
C24–N5, Å	1.303(5)	1.319(6)	1.303(7)
C6–Pt–N1, °	81.67(15)	81.53(12)	81.18(13)
N2–C6–N3, °	112.2(4)	113.8(3)	112.0(3)

Table S3. Geometrical parameters of the observed HBs in supramolecular dimers {**3A**}$_2$, {**3A’**}$_2$, {**3B**}$_2$.

**Dimer**	**Contact (X–H···Y)**	**d(X–H···Y), Å**	**d(X···Y), Å**	**∠(X–H···Y), °**
{**3A**}$_2$	C23-H23C···N6	2.466(4)	3.424(6)	165.7(2)
{**3B**}$_2$	C23A-H23E···N6A	2.929(4)	3.854(6)	157.8(2)
{**3A’**}$_2$	C23-H23C···N6	2.724(3)	3.573(4)	142.0(2)
	C1-H1···O1	2.739(3)	3.357(5)	123.3(2)

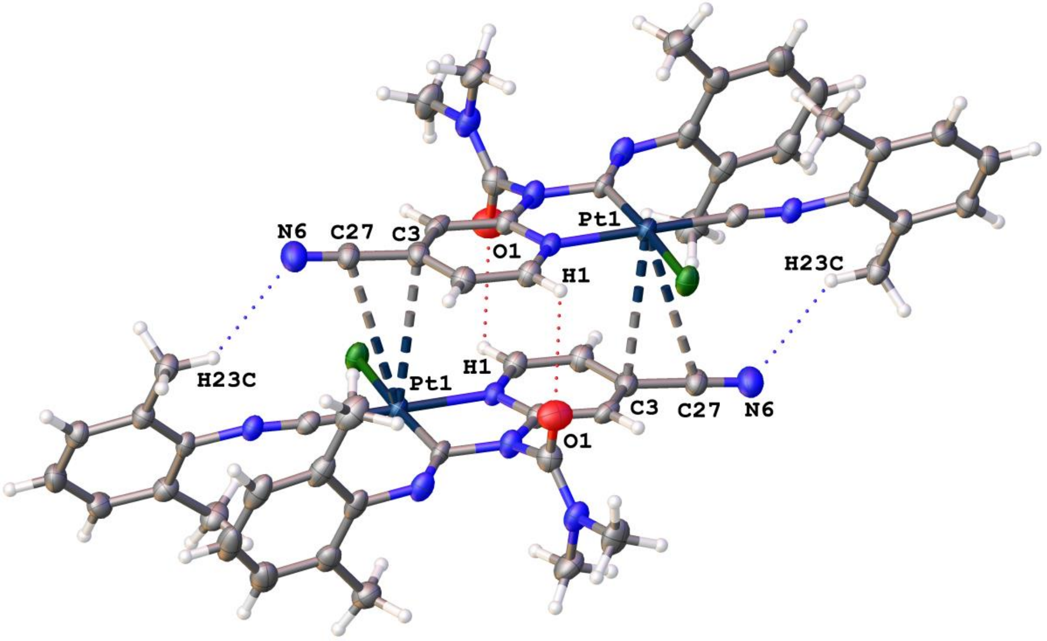

Figure S1. Structure of supramolecular dimer {**3A’**}$_2$.

2. Copies of NMR and HRMS spectra of complex 3

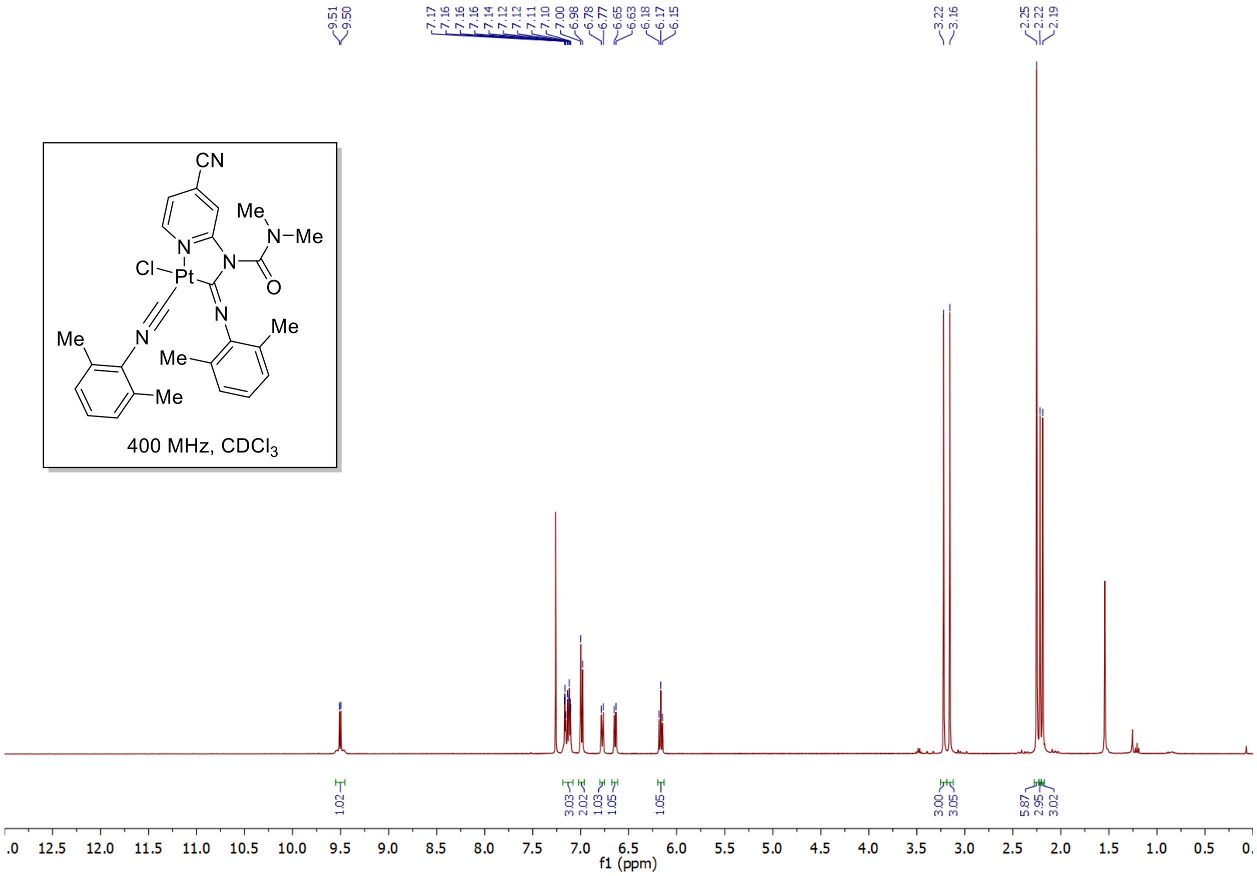

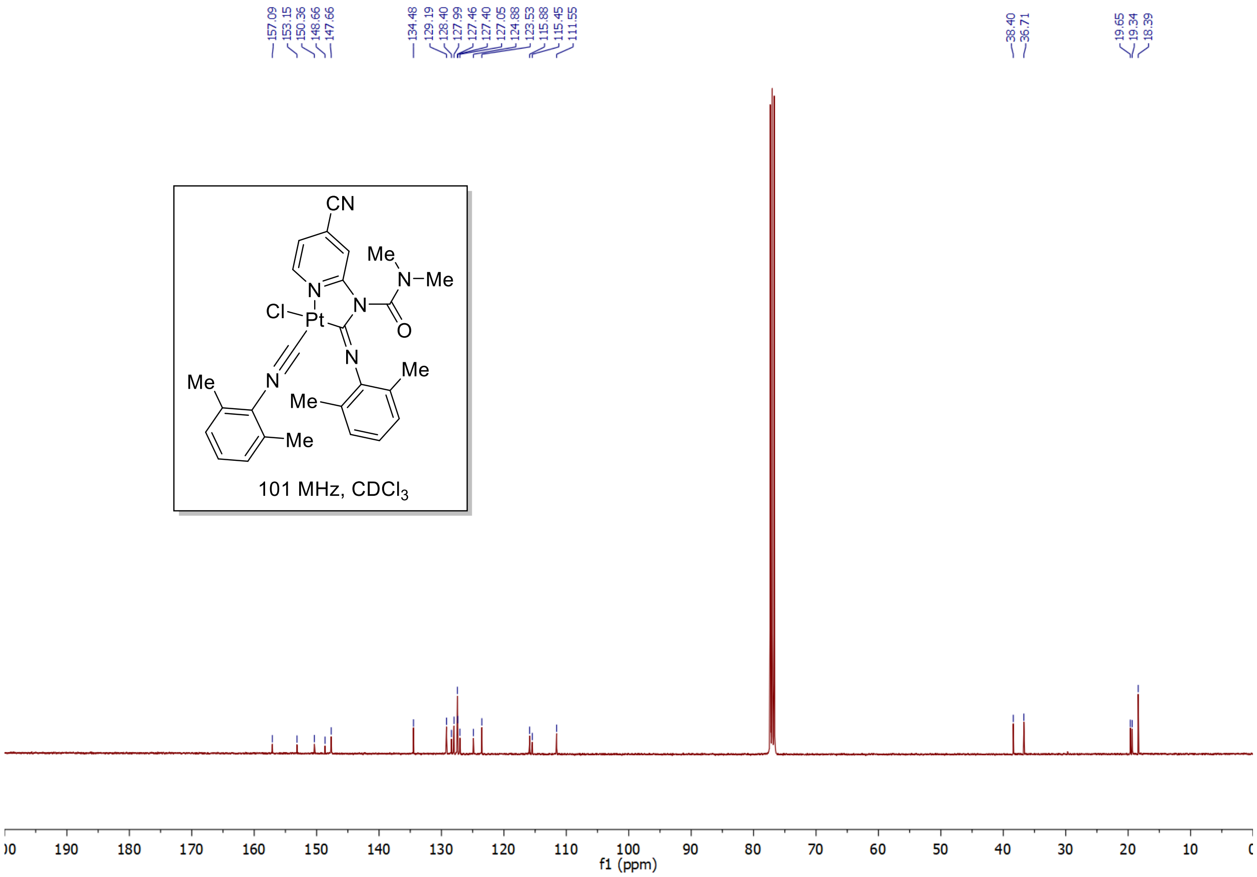

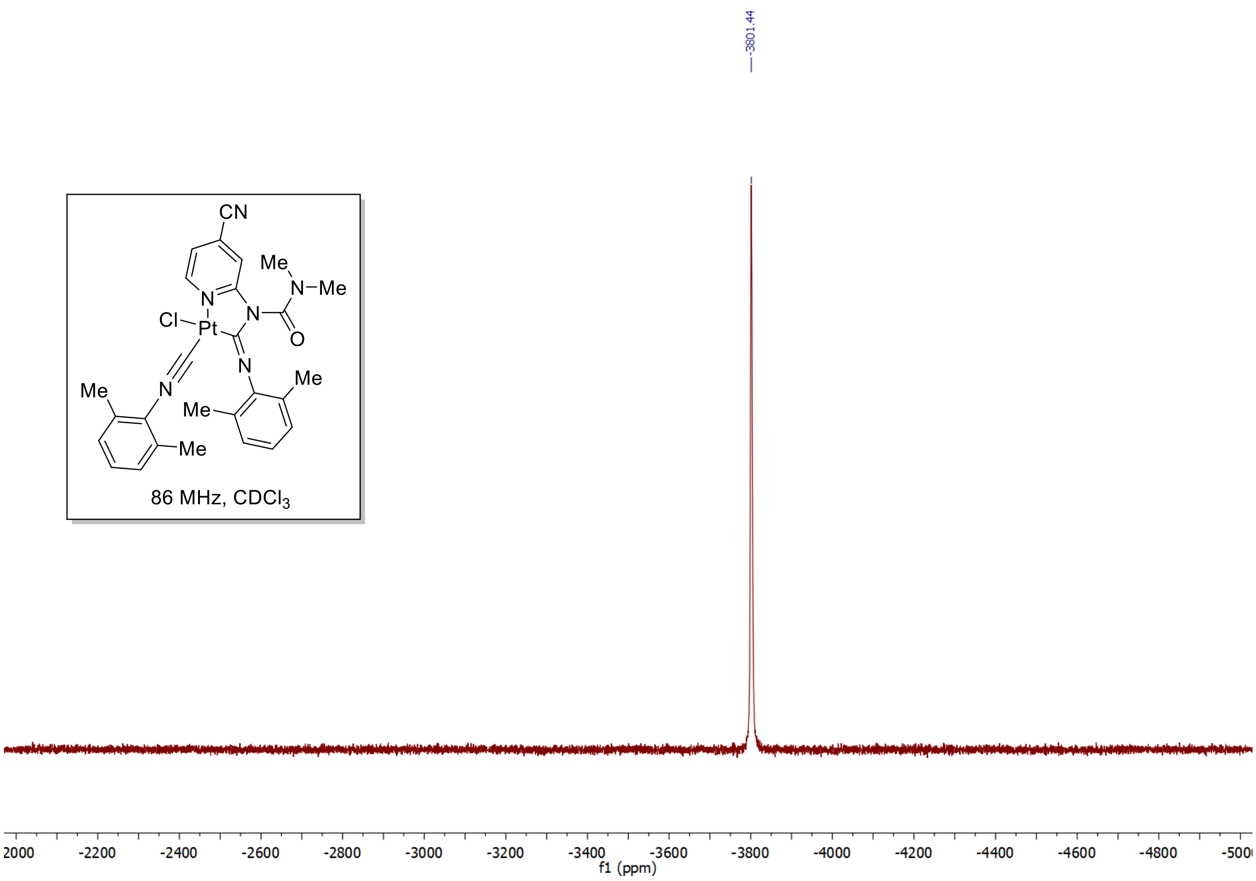
-3801.44
CN
Me
N–Me
N
N
O
Cl
Pt
N
N
Me
Me
Me
Me
86 MHz, $CDCl_3$
2000
-2200
-2400
-2600
-2800
-3000
-3200
-3400
-3600
-3800
-4000
-4200
-4400
-4600
-4800
f1 (ppm)

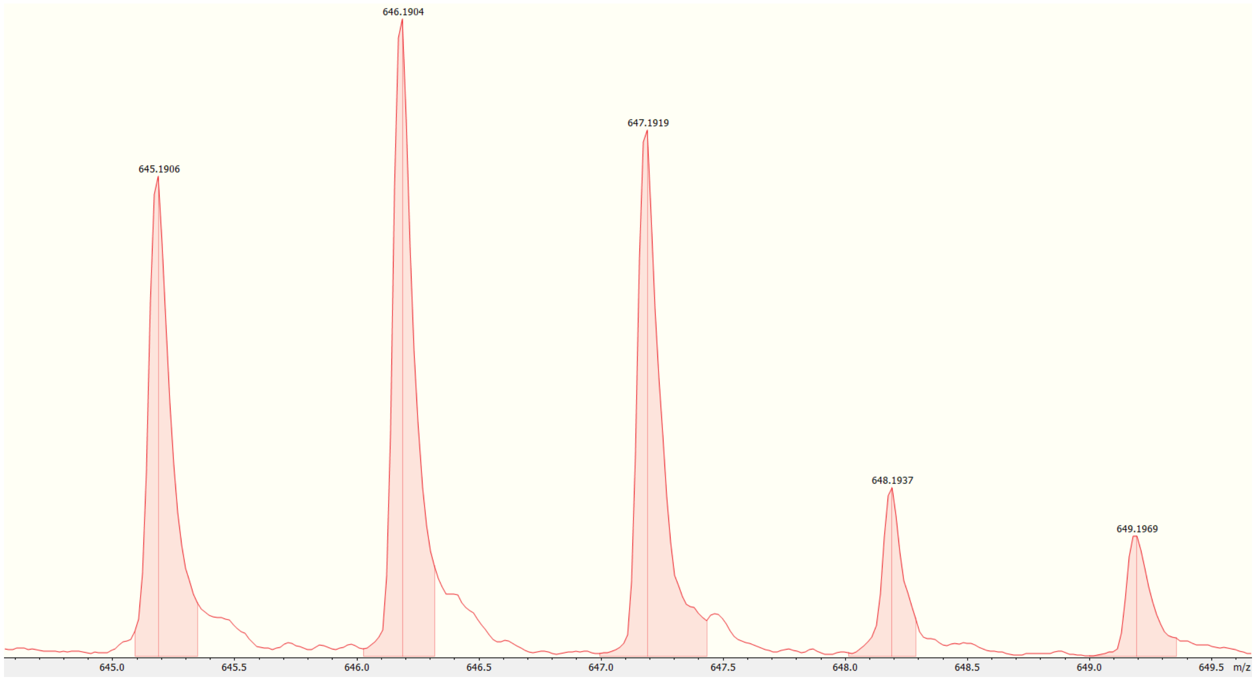
646.1904
647.1919
645.1906
648.1937
649.1969
645.0
645.5
646.0
646.5
647.0
647.5
648.0
648.5
649.0
649.5
m/z

3. Computational study

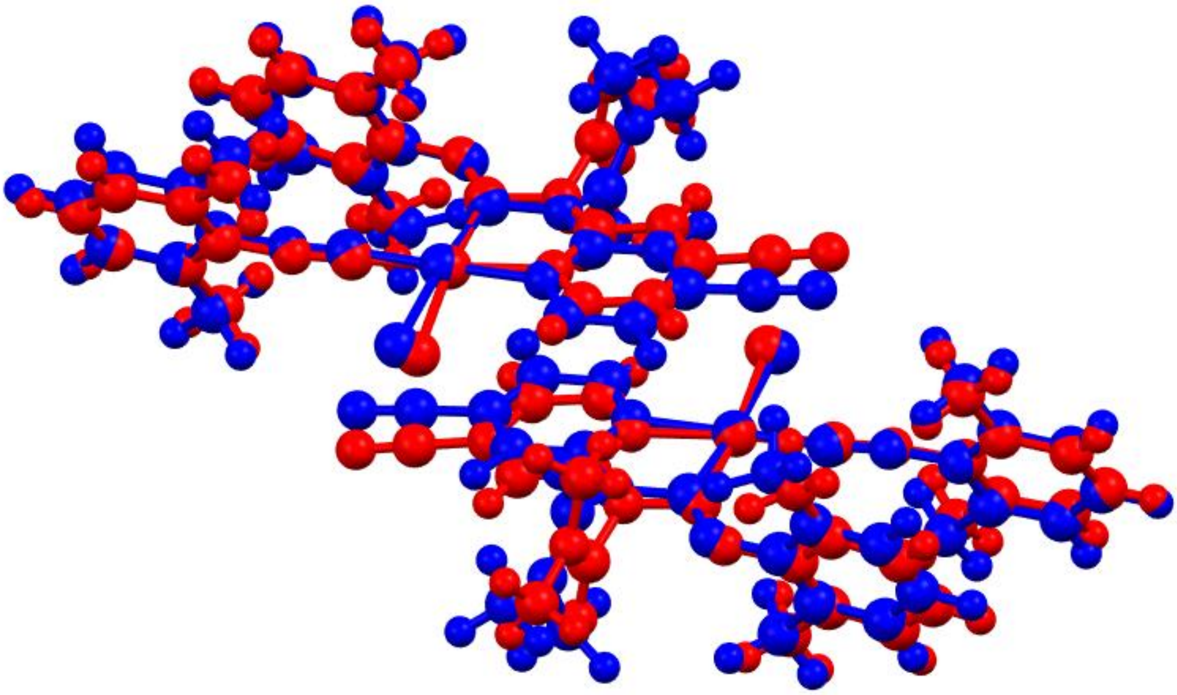

Figure S2. Overlay images for {**3A**}$_2$ of X-ray geometry (red) and its optimized (blue) computational clusters [**3A**]$_2$.

4. Cartesian coordinates for the studied molecules

Optimized geometries

Cartesian coordinate for **3A** (in Å)

Pt	-5.533809473	0.191634618	3.289121846
Cl	-5.722629195	2.539470232	3.073536815
N	-3.500929628	0.214363884	3.025596697
N	-8.571311271	0.285756221	3.480648518
N	-3.780539779	-2.034508362	3.354174805
O	-3.042137368	-3.765453035	2.026746425
N	-5.888588437	-2.786830361	3.705253014
N	-3.252346839	-4.150578331	4.259035762
C	-1.566450763	-1.178555150	2.794662805
H	-1.128043628	-2.171166601	2.769309545
C	-5.178108935	-1.755176332	3.486933613
C	-2.756032195	1.305683389	2.786588205
H	-3.311389506	2.244630523	2.788391786
N	1.737130233	-0.282450506	2.079655031
C	-2.937954076	-1.012176682	3.048842803
C	-1.399368621	1.228714010	2.548255072
H	-0.817859004	2.125332590	2.356675800
C	-9.937415414	0.398370314	3.531007854
C	-7.412195991	0.152411882	3.438466365
C	-10.664971985	0.251461094	2.336072085
C	-0.801112979	-0.045868963	2.547124144
C	0.602674821	-0.180502777	2.290563265
C	-10.541833914	0.650212931	4.775710974
C	-7.921629019	-2.607769125	5.029343289
C	-12.055000255	0.326429965	2.422718461
H	-12.647097091	0.210415775	1.515588303
C	-3.341575311	-3.402392128	3.141211809
C	-9.951277181	0.020179766	1.040494495

H	-10.660542828	-0.071500160	0.212381019
H	-9.349953202	-0.896677128	1.086257944
H	-9.263193692	0.845826835	0.815931248
C	-11.934773487	0.715626801	4.807586279
H	-12.433043258	0.903306417	5.758101997
C	-7.275501669	-2.791143439	3.789131303
C	-12.686063229	0.547875605	3.645673340
H	-13.772599670	0.600383201	3.691991606
C	-8.018675133	-3.059648781	2.620133283
C	-9.699474731	0.835671460	5.999687936
H	-9.079066221	-0.050132296	6.185165121
H	-10.322009625	1.014299040	6.881660531
H	-9.015041720	1.686262950	5.881912616
C	-3.599796120	-3.706583612	5.585164630
H	-2.806760701	-3.993645071	6.288089325
H	-4.543793337	-4.162140313	5.910495062
H	-3.715830455	-2.622178095	5.611113014
C	-9.315563213	-2.693905017	5.076481842
H	-9.821301154	-2.549560832	6.031876993
C	-2.952687960	-5.556792951	4.114116817
H	-3.827281947	-6.164667024	4.385161046
H	-2.115161160	-5.836661553	4.765973876
H	-2.686392969	-5.755966233	3.074345355
C	-10.062630213	-2.954043254	3.930239225
H	-11.148877037	-3.003868595	3.983961998
C	-7.119588209	-2.307097747	6.261025450
H	-6.418146703	-3.120587625	6.485382485
H	-7.770311825	-2.173139101	7.131858773
H	-6.518346045	-1.395983347	6.136215602
C	-9.410261725	-3.138411619	2.713392917
H	-9.988752427	-3.348269276	1.812890292

C	-7.303140437	-3.228899706	1.313074410
H	-6.882125765	-2.274392305	0.965359090
H	-7.977055199	-3.607345487	0.537026898
H	-6.453541347	-3.915952819	1.414306963

Cartesian coordinate for **3B** (in Å)

Pt	16.51466921207595	4.33330961605363	8.92455096437090
Cl	18.24181871577911	3.18629320915664	7.78843472525576
N	16.42778592733429	5.83796961181970	7.54449905373245
N	14.88453855853297	6.64344983164726	9.02073857832969
N	16.78256635462710	2.03129366177991	10.89625327063657
N	14.27292336791437	5.29347641248471	10.72312231053617
C	16.94271407976309	0.89834140921174	11.65715751954622
O	12.74381662835252	7.38471835316235	8.59098046594949
C	17.02629279491979	6.95647726918521	5.53897152942512
H	17.62395807392470	6.99464441317953	4.64019813106576
C	17.66553291483284	0.98981176688207	12.84793691498714
C	15.06333115718607	5.42358773744145	9.74149560408493
C	17.15524215495927	5.91607605645175	6.42266507956200
H	17.84686136382328	5.09491525098050	6.27322313102045
N	15.79017941323127	9.94452882697550	4.22068608471115
C	15.53719722928113	6.80197232882714	7.84387304656927
C	16.59570320431805	2.94272732595165	10.20647625756641
C	16.09410634387401	7.95656974795017	5.83617467977870
C	17.78838716343211	-0.16529980471055	13.60556892976439
H	18.34057551991467	-0.12644758748487	14.53749764965912
C	15.33901749657031	7.88898825824159	6.98903493215452
H	14.60016297239274	8.64156025206245	7.22087361512640
C	14.19089551323795	4.12377542910429	11.47571115872933
C	16.37441272819786	-0.29000216691047	11.19140007778456
C	18.27454765441078	2.28660687102772	13.26618082461005

H	17.51162894183956	3.06260672380247	13.35945566797565
H	18.99860943066608	2.63602605309489	12.52535623650671
H	18.78465520810532	2.18347334471878	14.22344174525389
C	17.21964664446781	-1.35626454633380	13.18331941327493
H	17.32546384941534	-2.24794315265268	13.78982006415309
N	13.87817148422700	8.32318041020828	10.31962378726326
C	14.77363375390048	4.07097607609771	12.74763039524589
C	15.92035588844617	9.05837083405570	4.94261019674569
C	16.52543167879221	-1.41699173285693	11.98559590807687
H	16.09250598501893	-2.35397478235573	11.65470695839991
C	13.44124518304052	3.04561645835686	10.98442256308625
C	15.63795199868181	-0.31825821949682	9.89410135927235
H	14.78349838637020	0.36288729718883	9.91981380946232
H	15.27533589353826	-1.32246222355485	9.67736415908368
H	16.28009256514446	0.00535818001184	9.07060915456886
C	14.62096687610561	2.91861162975555	13.50660898604601
H	15.08186587391256	2.86903522264484	14.48764211568724
C	13.30570683262645	1.91441391935617	11.77943791726745
H	12.72203918430995	1.07911576931318	11.40652152559528
C	13.72144752319411	7.46703692993281	9.29751159488246
C	12.80812486905459	3.11913519448389	9.63137143549492
H	13.56179477724646	3.08794682067359	8.83873933248150
H	12.26104653118399	4.05611482513629	9.50273557943107
H	12.12149157735711	2.28541474108998	9.48037464338343
C	13.89529004943994	1.84001976217702	13.02983512731692
H	13.79142470603697	0.94448118611685	13.63059971257998
C	15.55980376043194	5.23399079280851	13.26070073245427
H	16.38878480336652	5.48439387633338	12.59293251480054
H	15.96471308179490	5.02441802822838	14.25169023953843
H	14.93075959149323	6.12531651873425	13.33090036091357
C	15.04466022229652	8.40343742871521	11.16254906069013

H	14.82491705992763	8.00796259127338	12.15701347332018
H	15.34827744630511	9.44902704952298	11.26017651255719
H	15.86556491108450	7.83507819552682	10.73718371983287
C	12.73474782681347	9.09388240361675	10.75054193082236
H	11.93400085611991	8.97537087221852	10.02621517947795
H	13.00741876329644	10.14899736238453	10.82999875668253
H	12.39309144206089	8.74505607920132	11.72984259541719

Cartesian coordinate for [**3A**]$_2$ (in Å)

Pt	-5.589517000	0.179332000	3.168545000
Cl	-5.767295000	2.525301000	2.869154000
N	-3.534609000	0.221487000	3.195307000
N	-8.639254000	0.231837000	3.151570000
N	-3.848030000	-2.042138000	3.233861000
O	-3.370022000	-3.715727000	1.723997000
N	-5.946941000	-2.813139000	3.516663000
N	-3.107299000	-4.167398000	3.938088000
C	-1.576012000	-1.162666000	3.239078000
H	-1.124178000	-2.149622000	3.212978000
C	-5.241037000	-1.768710000	3.345799000
C	-2.769079000	1.324324000	3.196959000
H	-3.303935000	2.271641000	3.139166000
N	1.782023000	-0.201671000	3.481762000
C	-2.968429000	-1.006432000	3.209380000
C	-1.390573000	1.257849000	3.232648000
H	-0.805658000	2.171315000	3.201688000
C	-9.984205000	0.409796000	3.368225000
C	-7.477490000	0.118008000	3.155584000
C	-10.895965000	0.122312000	2.337945000
C	-0.792317000	-0.013650000	3.264023000
C	0.633413000	-0.128616000	3.357046000

C	-10.377245000	0.877222000	4.637090000
C	-7.884580000	-2.498568000	4.944737000
C	-12.251526000	0.292704000	2.623575000
H	-12.982821000	0.074400000	1.846478000
C	-3.436632000	-3.390010000	2.887507000
C	-10.416086000	-0.360598000	1.007405000
H	-11.249748000	-0.468883000	0.307212000
H	-9.921817000	-1.334627000	1.111011000
H	-9.671308000	0.311996000	0.567584000
C	-11.744031000	1.029379000	4.869268000
H	-12.077783000	1.386508000	5.842615000
C	-7.325238000	-2.817632000	3.688542000
C	-12.674446000	0.735279000	3.874824000
H	-13.737657000	0.859878000	4.074215000
C	-8.142531000	-3.226724000	2.616626000
C	-9.348156000	1.188306000	5.678949000
H	-8.771205000	0.291412000	5.938660000
H	-9.816782000	1.572586000	6.589529000
H	-8.628889000	1.934605000	5.316568000
C	-3.355478000	-3.821693000	5.317099000
H	-2.472837000	-4.056792000	5.924825000
H	-4.213443000	-4.385750000	5.707651000
H	-3.575222000	-2.757678000	5.412383000
C	-9.270770000	-2.573502000	5.097074000
H	-9.711013000	-2.320257000	6.061700000
C	-2.723607000	-5.536592000	3.676721000
H	-3.538099000	-6.225497000	3.941849000
H	-1.836291000	-5.795557000	4.266799000
H	-2.500784000	-5.644802000	2.613561000
C	-10.091866000	-2.967812000	4.043570000
H	-11.171001000	-3.013599000	4.178632000

C	-6.998827000	-2.088053000	6.084105000
H	-6.210851000	-2.832573000	6.255703000
H	-7.574785000	-1.977621000	7.008860000
H	-6.491023000	-1.135680000	5.879162000
C	-9.522911000	-3.301188000	2.817309000
H	-10.159404000	-3.625540000	1.993692000
C	-7.521681000	-3.527938000	1.285705000
H	-7.301791000	-2.597194000	0.742530000
H	-8.192163000	-4.125777000	0.659364000
H	-6.570086000	-4.059466000	1.406713000
Pt	0.000000000	0.000000000	0.000000000
Cl	0.211138000	-2.344273000	0.278021000
N	-2.054551000	-0.072672000	-0.018466000
N	3.050030000	0.000000000	0.000000000
N	-1.774081000	2.195659000	-0.039130000
O	-2.211832000	3.864452000	1.488445000
N	0.309384000	3.000992000	-0.328124000
N	-2.612800000	4.295763000	-0.708685000
C	-4.032844000	1.282861000	-0.064828000
H	-4.498903000	2.263121000	-0.038486000
C	-0.377814000	1.943936000	-0.159296000
C	-2.803859000	-1.186488000	-0.021686000
H	-2.254587000	-2.125648000	0.035710000
N	-7.374633000	0.277146000	-0.325852000
C	-2.638633000	1.147123000	-0.028504000
C	-4.183306000	-1.140186000	-0.060033000
H	-4.754901000	-2.062094000	-0.030242000
C	4.399304000	-0.144177000	-0.213855000
C	1.886386000	0.091233000	0.003080000
C	5.299161000	0.144416000	0.826574000
C	-4.799422000	0.122448000	-0.094302000

C	-6.225878000	0.217951000	-0.195167000
C	4.808903000	-0.578101000	-1.489215000
C	2.241516000	2.743646000	-1.772164000
C	6.659603000	0.010674000	0.544945000
H	7.382019000	0.230912000	1.329759000
C	-2.200983000	3.534090000	0.324716000
C	4.801231000	0.590134000	2.163580000
H	5.629211000	0.710139000	2.868596000
H	4.279547000	1.551279000	2.075316000
H	4.075371000	-0.112017000	2.588960000
C	6.179914000	-0.694616000	-1.716988000
H	6.526525000	-1.025283000	-2.695180000
C	1.686457000	3.029669000	-0.506461000
C	7.098508000	-0.398255000	-0.712270000
H	8.165249000	-0.494598000	-0.908439000
C	2.504578000	3.429630000	0.568136000
C	3.792104000	-0.891737000	-2.542319000
H	3.196153000	-0.003023000	-2.786803000
H	4.273644000	-1.246868000	-3.457992000
H	3.088604000	-1.661447000	-2.198548000
C	-2.409109000	3.962676000	-2.098221000
H	-3.337048000	4.119500000	-2.662549000
H	-1.622405000	4.594145000	-2.533375000
H	-2.104664000	2.920543000	-2.201653000
C	3.625321000	2.842388000	-1.932412000
H	4.062549000	2.615475000	-2.904952000
C	-3.048919000	5.644987000	-0.426165000
H	-2.271869000	6.371823000	-0.702305000
H	-3.959576000	5.868725000	-0.994478000
H	-3.252458000	5.734827000	0.642610000
C	4.447630000	3.227831000	-0.876553000

H	5.525072000	3.292030000	-1.017509000
C	1.351965000	2.341751000	-2.911497000
H	0.545940000	3.073003000	-3.054971000
H	1.919115000	2.265826000	-3.845139000
H	0.867230000	1.373866000	-2.723673000
C	3.882360000	3.528132000	0.359952000
H	4.519917000	3.844872000	1.185691000
C	1.887708000	3.695365000	1.908512000
H	1.687002000	2.750876000	2.435466000
H	2.552160000	4.292644000	2.541787000
H	0.926269000	4.212510000	1.803739000

Cartesian coordinate for [**3B**]$_2$ (in Å)

Pt	16.705157000	4.999167000	8.580572000
Cl	18.408864000	3.920626000	7.297961000
N	16.329587000	6.410230000	7.144849000
N	14.976510000	7.225362000	8.801943000
N	17.226639000	2.821817000	10.658272000
N	14.488212000	5.772593000	10.480054000
C	17.343662000	1.612838000	11.305275000
O	12.830442000	8.041983000	8.581899000
C	16.513677000	7.385652000	4.980459000
H	16.945817000	7.375691000	3.984635000
C	17.763519000	1.566459000	12.644724000
C	15.241096000	5.997151000	9.481978000
C	16.855886000	6.427137000	5.908183000
H	17.555825000	5.619091000	5.703045000
N	14.834526000	10.182051000	3.675321000
C	15.446804000	7.353702000	7.530019000
C	16.985031000	3.695263000	9.920521000
C	15.579225000	8.367240000	5.365317000

C	17.815798000	0.309567000	13.250596000
H	18.131599000	0.241305000	14.290747000
C	15.046538000	8.367164000	6.643651000
H	14.319768000	9.109428000	6.950884000
C	14.438248000	4.568498000	11.169565000
C	16.997647000	0.464185000	10.564533000
C	18.157214000	2.805273000	13.384782000
H	17.558910000	3.665973000	13.070335000
H	19.210829000	3.044531000	13.192105000
H	18.027692000	2.669803000	14.463314000
C	17.472919000	-0.843312000	12.549858000
H	17.520634000	-1.811949000	13.044943000
N	14.095349000	8.802571000	10.313034000
C	14.871336000	4.523550000	12.509290000
C	15.170375000	9.376841000	4.436932000
C	17.071441000	-0.765098000	11.217720000
H	16.806564000	-1.669653000	10.672057000
C	13.869450000	3.429572000	10.556552000
C	16.548364000	0.588216000	9.141994000
H	15.612843000	1.160712000	9.079961000
H	16.376755000	-0.397235000	8.699636000
H	17.285903000	1.123243000	8.529053000
C	14.769614000	3.321866000	13.212021000
H	15.113713000	3.282669000	14.245438000
C	13.791284000	2.246432000	11.295962000
H	13.357190000	1.362414000	10.828508000
C	13.855714000	8.047471000	9.224879000
C	13.345310000	3.498080000	9.151709000
H	14.160789000	3.522183000	8.415350000
H	12.756782000	4.410938000	8.995989000
H	12.713905000	2.631588000	8.929621000

C	14.244262000	2.181364000	12.610669000
H	14.187052000	1.246116000	13.164575000
C	15.447050000	5.757009000	13.135550000
H	16.391835000	6.046417000	12.653723000
H	15.639871000	5.610673000	14.203303000
H	14.762535000	6.605552000	13.010983000
C	15.357318000	8.896770000	11.003749000
H	15.200068000	8.769617000	12.081769000
H	15.825581000	9.873241000	10.820548000
H	16.037821000	8.117778000	10.664245000
C	13.023040000	9.622639000	10.825302000
H	12.143001000	9.487812000	10.193820000
H	13.314438000	10.681537000	10.819528000
H	12.778947000	9.330959000	11.855715000
Pt	18.922392000	9.230311000	6.887176000
Cl	17.219187000	10.308702000	8.169983000
N	19.298216000	7.819064000	8.322868000
N	20.651224000	7.004164000	6.665562000
N	18.400894000	11.408073000	4.809741000
N	21.139261000	8.456957000	4.987453000
C	18.283378000	12.617465000	4.163563000
O	22.797039000	6.186731000	6.885100000
C	19.114318000	6.843217000	10.487107000
H	18.682198000	6.853064000	11.482934000
C	17.864117000	12.664457000	2.823925000
C	20.386494000	8.232397000	5.985629000
C	18.771885000	7.801781000	9.559512000
H	18.071655000	8.609541000	9.764788000
N	20.794036000	4.047311000	11.792193000
C	20.181034000	6.875686000	7.937528000
C	18.642399000	10.534498000	5.547385000

C	20.048951000	5.861867000	10.102100000
C	17.811077000	13.921732000	2.218929000
H	17.495717000	13.990493000	1.178678000
C	20.581510000	5.862149000	8.823700000
H	21.308464000	5.120080000	8.516434000
C	21.189098000	9.660944000	4.297760000
C	18.628103000	13.765839000	4.905316000
C	17.471744000	11.425823000	2.082885000
H	18.070603000	10.565396000	2.397013000
H	16.418215000	11.185608000	2.274889000
H	17.601644000	11.561971000	1.004477000
C	18.152706000	15.074371000	2.920678000
H	18.104448000	16.043331000	2.426276000
N	21.531583000	5.426989000	5.154012000
C	20.755916000	9.705670000	2.958049000
C	20.458158000	4.852506000	11.030575000
C	18.553632000	14.995519000	4.252934000
H	18.817465000	15.899910000	4.799375000
C	21.758024000	10.799960000	4.910466000
C	19.076728000	13.641205000	6.328008000
H	20.012771000	13.069580000	6.390111000
H	19.247158000	14.626487000	6.771197000
H	18.339344000	13.105031000	6.940135000
C	20.857792000	10.907160000	2.255035000
H	20.513673000	10.946152000	1.221619000
C	21.836280000	11.982937000	4.170804000
H	22.270457000	12.867022000	4.638052000
C	21.771699000	6.181832000	6.242233000
C	22.282279000	10.731646000	6.315285000
H	21.466839000	10.707342000	7.051678000
H	22.871028000	9.818929000	6.471019000

H	22.913485000	11.598293000	6.537324000
C	21.383299000	12.047760000	2.856096000
H	21.440649000	12.982853000	2.301946000
C	20.179959000	8.472197000	2.332041000
H	19.235870000	8.182230000	2.814887000
H	19.985853000	8.618829000	1.264561000
H	20.864931000	7.623826000	2.455420000
C	20.269357000	5.333073000	4.463728000
H	20.426414000	5.459466000	3.385597000
H	19.800583000	4.356988000	4.647705000
H	19.589374000	6.112680000	4.802876000
C	22.603460000	4.606523000	4.641472000
H	23.483928000	4.741544000	5.272314000
H	22.311974000	3.547646000	4.648001000
H	22.846859000	4.897632000	3.610740000
